# Cerebral Hemodynamics and Systemic Endothelial Function Are Already Impaired in Well-Controlled Type 2 Diabetic Patients, with Short-Term Disease

**DOI:** 10.1371/journal.pone.0083287

**Published:** 2013-12-31

**Authors:** Paola Palazzo, Paola Maggio, Riccardo Altavilla, Alessandra Di Flaviani, Ilaria Giordani, Ilaria Malandrucco, Fabiana Picconi, Francesco Passarelli, Patrizio Pasqualetti, Matilde Ercolani, Fabrizio Vernieri, Simona Frontoni

**Affiliations:** 1 Department of Neurology, Campus Bio-Medico University, Rome, Italy; 2 Department of Neurology, S. Giovanni Calibita Fatebenefratelli Hospital, Rome, Italy; 3 Endocrinology, Diabetes and Metabolism, S. Giovanni Calibita Fatebenefratelli Hospital, University of Rome Tor Vergata, Rome, Italy; 4 SeSMIT, Service for Medical Statistics & IT, Fatebenefratelli Association for Research, Isola Tiberina, Rome, Italy; Charité University Medicine Berlin, Germany

## Abstract

**Objective:**

Impaired cerebral vasomotor reactivity (VMR) and flow-mediated dilation (FMD) were found in selected subgroups of type 2 diabetes mellitus (T2DM) patients with long-term disease. Our study aimed to evaluate cerebral hemodynamics, systemic endothelial function and sympatho-vagal balance in a selected population of well-controlled T2DM patients with short-term disease and without cardiac autonomic neuropathy (CAN).

**Research Design and Methods:**

Twenty-six T2DM patients with short-term (4.40±4.80 years) and well-controlled (HbA1C = 6.71±1.29%) disease, without any complications, treated with diet and/or metformin, were consecutively recruited. Eighteen controls, comparable by sex and age, were enrolled also.

**Results:**

FMD and shear rate FMD were found to be reduced in T2DM subjects with short-term disease (8.5% SD 3.5 and 2.5 SD 1.3, respectively) compared to controls (15.4% SD 4.1 and 3.5 SD 1.4; p<.001 and p<.05). T2DM patients also displayed reduced VMR values than controls (39.4% SD 12.4 vs 51.7%, SD 15.5; p<.05). Sympatho-vagal balance was not different in T2DM patients compared to healthy subjects. FMD and shear rate FMD did not correlate with VMR in T2DM patients or in controls (p>.05).

**Conclusions:**

In well-controlled T2DM patients with short-term disease cerebral hemodynamics and systemic endothelial function are altered while autonomic balance appeared to be preserved.

## Introduction

Type 2 diabetes mellitus (T2DM) is associated with an increased risk of cardiovascular and cerebrovascular diseases with high mortality and disability. Cerebral vasomotor reactivity (VMR), one of the most accurate markers of cerebral hemodynamics, has been shown to be impaired in subjects with carotid artery steno-occlusive disease and associated with an increased risk of ischemic events [1], [2], [3]. Among the factors associated with cerebrovascular events, endothelial dysfunction, evaluated as brachial artery flow-mediated dilation (FMD), and cardiac autonomic neuropathy (CAN) seem to play a major role [4], [5], [6].

Impaired VMR and FMD were found in selected subgroups of T2DM patients with long-term disease [7] or in type 1 diabetic patients with nephropathy [8]. However, VMR was normal in unselected patients [9], and no data are available in well-controlled patients with short-term disease. These controversial results are likely due to the heterogeneity of the studied populations, since it is well known that the degree of metabolic control and the presence of diabetic complications strongly contribute to determine the different phenotypes of type 2 diabetic patients. Particularly, CAN seems to play a fundamental role, since autonomic innervations are known to regulate systemic vascular endothelial function, evaluated by FMD [10], [11], and a role, although still unclear, of the vegetative nervous system on cerebral vasomotor reactivity has been demonstrated [12], [13]. Spectral analysis of heart rate variability (HRV) is a non-invasive tool used to study autonomic nervous system activity, in vivo. High frequency (HF) component is mainly influenced by vagal activity and low frequency both by sympathetic and parasympathetic ones, thus the LF/HF ratio is considered a reliable index of sympatho-vagal balance in humans.

This study aimed to evaluate cerebral hemodynamics, systemic endothelial function and sympatho-vagal balance in a selected population of well-controlled T2DM patients with short-term disease and without overt CAN.

## Materials and Methods

### Ethics statement

The experimental protocol was approved by the Biomedical Ethics Committee of San Giovanni Calibita Fatebenefratelli Hospital of Rome, and all subjects signed a written consent form before the screening tests were performed. All clinical investigation were conducted according to the principles expressed in the Declaration of Helsinki.

Twenty-six T2DM patients with short-term (duration of diabetes <10 years), good metabolic control (HbA1c <8%), and normo-albuminuria (albumin excretion rate, AER, <30 mg/gr urinary creatinine), treated with diet and/or metformin, were consecutively recruited from those followed at the Department of Endocrinology and Diabetes, S. Giovanni Calibita Fatebenefratelli Hospital of Rome. Eighteen age- and sex-matched controls were enrolled also.

Exclusion criteria were: a medical history of any vascular ischemic event; history of arterial hypertension or anti-hypertensive treatment; documented CAN; micro and macroalbuminuria; proliferative retinopathy; estro-progestinic therapy; vasoactive drugs; carotid artery stenosis >40% according to ECST criteria [14]; vertebral and proximal subclavian artery abnormalities; differences between right and left brachial artery blood pressures; brachial arteries blood flow pattern alterations; poor insonation of the middle cerebral artery (MCA) through transtemporal bone windows; intracranial artery abnormalities.

All control subjects underwent OGTT to exclude diabetes and impaired glucose tolerance.

All subjects underwent a careful clinical evaluation and the European Society of Cardiology (ESC) score was calculated. Color-coded duplex sonography of the neck vessels (ACUSON C512 Sequoia, Siemens, Germany) and transcranial Doppler (DWL MultiDop X4, Elektronische Systeme GmbH, Germany) evaluation were performed on all subjects. A high-resolution B-mode system with linear ultrasound transducers at 9 MHz was used; depth of focus, frame rate and gain settings were adjusted in order to obtain optimal image quality. In each subject, intima-media thickness (IMT) was measured, by a single experienced ultrasonographer, on the far wall of the distal common carotid artery, 1 cm proximal to the bifurcation, with the mean-value calculated on a 10-mm segment of the artery. In order to improve reliability and reproducibility of measurements, semiautomatic software was used [15].

In order to exclude CAN, cardiac autonomic function was assessed in T2DM patients using the battery of cardiovascular tests proposed by Ewing [16] and recommended by the Toronto Consensus [17]. Blood and urinary samples were obtained for the determination of Albumin Excretion Rate (AER), cholesterol, triglycerides and HbA1C.

HbA1c was analysed by High Performance Liquid Chromatography (HPLC, VARIANT 2, BIORAD Laboratories, Munich, Germany), with intra- and inter-assay coefficients of variation of 0.46–0.77% and 0.69–0.91%, respectively. Total cholesterol, HDL-cholesterol and triglycerides were determined by an enzymatic colorimetric test (Cobas, Roche Diagnostic, Indianapolis, USA). AER was determined by immunoturbimetric-assay (Tina-quant, Cobas, Roche Diagnostic, Indianapolis, USA).

The entire study protocol was performed between 8 and 9 AM in a quiet, temperature controlled room (22°C to 24°C). The tests were conducted after a 12-hour overnight fast. No caffeine, theine, or alcohol were permitted 12 hours preceding the study; smokers refrained from smoking for the same time period.

FMD was measured as brachial artery diameter changes induced by transient ischemia, according to guidelines [4]. All examinations were performed by a single experienced vascular sonographer, who was unaware of the subjects' clinical background, using an ultrasound system (ACUSON C512 Sequoia, Siemens, Germany) with a broadband 8–14 MHz transducer. In our ultrasound laboratory the coefficient of variation for FMD repeated measurements was 15%.

With subjects in a supine position, the right brachial artery was scanned over a longitudinal section, 3–5 cm above the elbow. Depth and gain settings were optimized to identify the lumen-to-vessel wall interface. A cuff was placed around the arm, proximally to the transducer, and inflated to 50 mmHg above systolic blood pressure for 5 minutes. After the cuff was deflated, the FMD was assessed by measuring the change in brachial artery diameter after 50, 60 and 70 seconds of reactive hyperemia and compared with baseline measurements. Arterial diameter was measured as the distance between intima layers from the far to near vessel wall. The mean diameter was calculated from three measurements of arterial diameter performed at end-diastole incident with the R wave on a continuously recorded ECG. The response of the vessel diameter to reactive hyperemia (FMD) was expressed as a percentage change relative to the diameter before cuff inflation. To avoid the possible bias due to basal diameter and peak flow velocity induced by reactive hyperemia, FMD raw values were corrected for flow velocity and basal diameter. Peak shear rate, expressed as peak flow velocity divided by baseline diameter, was calculated to quantify the FMD stimulus in each subject and FMD responses were normalized by dividing the maximal percentage change in diameter by the peak shear rate [18], thus shear rate FMD was assessed.

After undergoing FMD assessment, each subject's VMR was also evaluated, with an at-rest 30-minute interval between the two tests.

VMR to hypercapnia was assessed by means of a CO_2_ inhalation test as elsewhere described [1]. Two trans-cranial Doppler (TCD) dual 2-MHZ transducers, fitted on a headband and placed on the temporal bone windows, were used to obtain bilateral continuous measurement of mean flow velocity (MFV) in the middle cerebral arteries (MCAs) insonated at a depth of 50±4 mm. During the experiment, end-tidal expiratory CO_2_ was measured by means of a capnometer (Drager Capnodig, Lübeck, Germany). The maximal vasodilatory range, or reactivity to 7% CO_2_, was determined by the percentage increase in MCA flow velocity recorded during the administration of 7% CO_2_, according to the following formula:
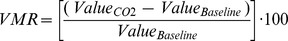
Twenty-one subjects (10 T2DM patients and 11 controls) also underwent a 5-minute ECG monitoring. Using fast Fourier transformation, fluctuations in RR interval widths were transformed into a frequency waveform that depicted periodic oscillations in sympathetic and parasympathetic functions. The frequency domain variables included total power (0.01 to 0.40 Hz), very low frequency (0.01 to 0.04 Hz), low frequency (LF 0.04 to 0.15 Hz) and high frequency (HF 0.15 to 0.40 Hz). To better define the role of the sympathetic-parasympathetic system, normalized LF (LFN) and HF (HFN) and LF/HF ratio were calculated. LFN was calculated according to the formula LFN = [LF/(Tot Power−VLF)]*100; HFN was calculated according to the formula HFN = [HF/(Tot Power−VLF)]*100.

### Statistical analysis

VMR inter-hemispheric concordance was measured by using the Intra-Class-Correlation index (ICC). The systematic difference between the two sides was evaluated with paired t-test, which showed no differences between right and left VMR. This permitted the use of the average of right- and left-sided VMR as a reliable measure of vasomotor reactivity.

All continuous variables were assessed for normal distribution of the values. Since their distribution was not different from gaussianity assumptions, independent samples Student's t-test was performed to compare continuous variables between groups. Chi-square test was used to compare categorical variables, Mann-Whitney test to compare ordinal data.

To study the correlation between FMD, shear rate FMD and VMR, Pearson's r correlation was used. Since inference on this index relies on specific assumptions (bivariate gaussianity of variables and robustness with respect to the effects of eventual outliers), we evaluated the departure from gaussianity and re-ran the procedure even after deleting the outliers.

Statistical analyses were performed with the SPSS 19 software (SPSS Inc).

## Results

Demographic characteristics and vascular risk factors are shown in Table 1. Controls and T2DM patients were comparable for common vascular risk factors except for higher HDL cholesterol and lower triglyceride levels in controls. In addition, six out of 18 control subjects were overweight. However, no one was obese, and waist circumference was significantly increased in T2DM patients compared to controls (p<.05). As expected, due to the presence of diabetes, median ESC score and IMT were significantly higher in T2DM than in controls (Table 1). In T2DM, mean duration of diabetes was 4.40±4.80 years, HbA1C 6.71±1.29%, AER 7.1±7.6 mg/gr of urinary creatinine.

**Table 1:** Demographic characteristics and vascular risk factors of the study population.^a^

Variable	T2DM patients (n = 26)	Controls (n = 18)	p
Age, years	58 (9)	54 (8)	.08
Male gender	11 (42)	4 (22)	.167
BMI	27 (3)	25 (3)	.052
Waist circumference, cm	93 (10)	81 (5)	**<.05**
Smoking	11 (42)	3 (17)	.073
Systolic Blood Pressure, mmHg	128 (11)	115 (13)	**<.001**
Diastolic Blood Pressure, mmHg	80 (6)	77 (7)	.07
Total Cholesterol, mg/dl	202 (34)	218 (33)	.171
LDL Cholesterol, mg/dl	126 (27)	131 (31)	.595
HDL Cholesterol, mg/dl	51 (12)	71 (14)	**<.001**
Triglycerides, mg/dl	121 (45)	73 (28)	**<.05**
Familiar history of cerebrovascular disease	17 (65)	8 (44)	.168
ESC score	1.5 (0–8)	0 (0–3)	**.003**
Mean IMT	0.7 (0.5–1.3)	0.6 (0.5–0.9)	**.004**

BMI: body mass Index; IMT: intima media thickness. Significant interactions are evidenced in bold.

^a^ For continuous variables, values were expressed as mean ± SD; for categorical variables, percent were used. ESC score and IMT were expressed as median (min-max range).

**Figure 1:**
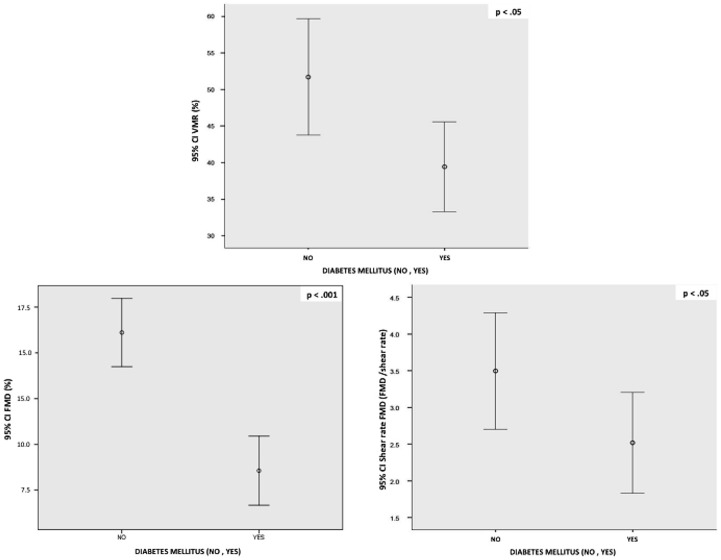
FMD, shear rate FMD and VMR in diabetic and control groups. Legend: FMD (Flow-Mediated Dilation, %); shear rate FMD (%); VMR (Cerebral Vasomotor Reactivity, %), LF/HF ratio (Low Frequency/High Frequency ratio). Data are expressed as mean ± SD.

FMD and shear rate FMD were found to be reduced in T2DM subjects (8.5% SD 3.5 and 2.5 SD 1.3, respectively) compared to controls (15.4% SD 4.1 and 3.5 SD 1.4; p<.001 and p<.05. Figure 1).

No systematic differences between right and left-side values of the CO_2_-induced VMR were observed, with a high correlation showed between the 2 sides (in both cases r = 0.8, p<0.001). Therefore, the average of right and left values was considered in our statistical analysis.

VMR also appeared to be significantly reduced in T2DM patients (39.4% SD 12.4) compared to controls (51.7%, SD 15.5) (p<.05, Figure 1).

After correcting for HDL cholesterol levels, smoking habits and BMI, the above mentioned differences were confirmed.

LF/HF ratio (1.2, SD 0.7 vs 1.5, SD 0.9, p = .442) and the single LFN (49.6 SD 16 vs 54.8 SD 15.2, p = .455) and HFN components (50.3 SD 16 vs 45.1 SD 15.2, p = .455) were similar in T2DM compared to controls.

No correlation between FMD and shear rate FMD respectively with VMR was found, both in T2DM patients and in controls (p>.05).

## Discussion

In our study, well-controlled T2DM patients with short-term disease already displayed reduced VMR values when compared to age- and sex-matched controls. It is, however, worth noting that VMR value was >20% in all patients, which is considered the cut off between normal and altered reactivity to 7% CO_2_ [19].

Data on cerebrovascular reactivity in diabetes are few and unclear, some previous studies showing reduced VMR in both type 1 and type 2 diabetic patients with long-term disease and diabetes complications [7], [8], [20], inversely related to the duration of diabetes [7] and albuminuria [20]. On the contrary, a population-based study demonstrated normal VMR in unselected patients with T2DM [9]. To our knowledge this is the first study assessing VMR in a well-controlled T2DM group with short-term disease, and normoalbuminuria. Even at this initial stage of the disease, without clinically relevant vascular and autonomic complications, cerebral hemodynamics is slightly still significantly altered.

This early cerebral hemodynamic dysfunction could be one of the main pathophysiological mechanisms underlying the increased risk of ischemic and atrophic brain damage as well as cognitive impairment observed in patients with diabetes [21], [22].

Moreover, we also found that endothelial function, evaluated as FMD, was reduced in our group of diabetic patients compared with controls. These data are consistent with previous studies demonstrating impaired endothelial function in type 2 diabetic patients. It should be underlined that these previous data were obtained in diabetic patients with chronic complications [23], or poor metabolic control [24]. The mechanisms of glucose-mediated endothelial dysfunction [25] include reduced NO bioavailability due to increased reactive oxygen species formation [26], and glucose auto-oxidation [27], [28]. Our patients with short-term disease were accurately selected with optimal metabolic control, in order to exclude the impact of marked hyperglycemia on endothelial function. However, clinical characteristics of metabolic syndrome (low HDL cholesterol, high triglyceride, increased waist circumference, increased BP), suggestive of insulin resistance, were significantly increased in the group of diabetic patients, when compared to controls. Therefore, our finding of impaired VMR and FMD in this selected population of T2DM patients strongly suggests that insulin-resistance has an impact on vascular function, even in the absence of metabolic derangement. Activation of oxidative stress pathways, by increased glucose variability, as previously demonstrated [29], might also have a negative impact on endothelial function.

The exclusion of CAN and the observation of a preserved sympatho-vagal balance, proven by LF/HF values within the normal range according to age [30], allowed us to rule out the role of impaired autonomic innervation of the cerebral vessels in early alterations of cerebrovascular reactivity observed in our population.

The lack of correlation between FMD and VMR confirms previous data obtained in patients with lacunar infarction [31], and in subjects with no history of vascular disease [32], suggesting different responses in different vascular districts. Similarly, several experimental studies evaluating the role of endothelial function mediators on cerebrovascular reactivity led to discordant results [33], [34], [35]. In particular, the role of NO in mediating the cerebral vasodilatory response to hypercapnia is still unclear, since some papers reported an inhibition by L-arginine analogues [33], [35], thus suggesting a role for NO, while others found no effects of NO-synthase inhibitor on cerebrovascular reactivity to CO_2_, assessed by internal and common carotid artery volume flow and middle cerebral artery flow velocity [34].

The main limitation of our study is the relatively small sample size. However, the presence of statistical differences between the two groups in both cerebral and peripheral vascular function reinforces our hypothesis that diabetes causes subclinical vascular complications also in the presence of optimal metabolic control and relatively short duration of the disease. This slight still significant alteration is likely to be due to initial endothelial dysfunction, and not to altered autonomic control of vessel smooth muscle cell tone.

Endothelium independent vasodilation was not evaluated in the present study, due to our hospital policy. This measure could have helped us to more accurately assess vascular smooth muscle function.

Another limitation of our study is the presence of a difference in the number of smokers between diabetic subjects and controls. However, this difference was not statistically significant, and, also after correcting for this variable, our results were confirmed.

A further limitation of this study is that neuroimaging was not available for the majority of our subjects. Therefore, we can exclude only clinically relevant vascular episodes, while silent cerebrovascular events cannot be ruled out.

In conclusion, the observation of impaired cerebral hemodynamics and systemic endothelial function in T2DM patients with well-controlled disease and preserved autonomic balance, but with clinical features of metabolic syndrome strongly suggests that factors other than chronic hyperglycemia (insulin-resistance, glucose variability) play a role in vascular dysfunction even in the absence of marked metabolic derangement. The observation of an impaired cerebrovascular reactivity in patients with T2DM is of particular interest, since it could be responsible for the increased risk of stroke and silent cerebral ischemia observed in patients with diabetes mellitus. Long-term prospective studies should be performed in order to evaluate the clinical course of cerebrovascular impairment and endothelial dysfunction in the natural history of diabetic disease.
